# Pancreatic Transdifferentiation and Glucose-Regulated Production of Human Insulin in the H4IIE Rat Liver Cell Line

**DOI:** 10.3390/ijms17040534

**Published:** 2016-04-08

**Authors:** Binhai Ren, Chang Tao, Margaret Anne Swan, Nichole Joachim, Rosetta Martiniello-Wilks, Najah T. Nassif, Bronwyn A. O’Brien, Ann M. Simpson

**Affiliations:** 1 School of Life Sciences and Centre for Health Technologies, University of Technology Sydney, P.O. Box 123, Broadway, 2007 Sydney, NSW, Australia; Binhai.Ren@uts.edu.au (B.R.); Chang.Tao@sswahs.nsw.gov.au (C.T.); Rosetta.Martiniello-Wilks@uts.edu.au (R.M.-W.); Najah.Nassif@uts.edu.au (N.T.N.); Bronwyn.Obrien@uts.edu.au (B.A.O.); 2 School of Medical Sciences (Anatomy & Histology) and Bosch Institute, University of Sydney, 2006 Sydney, NSW, Australia; swan@anatomy.usyd.edu.au (M.A.S.); nichole.joachim@gmail.com (N.J.)

**Keywords:** diabetes, gene therapy, H4IIE cells, regulated insulin secretion, insulin storage, secretory granules, liver cells, β cell transcription factors, furin-cleavable human insulin

## Abstract

Due to the limitations of current treatment regimes, gene therapy is a promising strategy being explored to correct blood glucose concentrations in diabetic patients. In the current study, we used a retroviral vector to deliver either the human insulin gene alone, the rat *NeuroD1* gene alone, or the human insulin gene and rat *NeuroD1* genes together, to the rat liver cell line, H4IIE, to determine if storage of insulin and pancreatic transdifferentiation occurred. Stable clones were selected and expanded into cell lines: H4IIEins (insulin gene alone), H4IIE/ND (*NeuroD1* gene alone), and H4IIEins/ND (insulin and *NeuroD1* genes). The H4IIEins cells did not store insulin; however, H4IIE/ND and H4IIEins/ND cells stored 65.5 ± 5.6 and 1475.4 ± 171.8 pmol/insulin/5 × 10^6^ cells, respectively. Additionally, several β cell transcription factors and pancreatic hormones were expressed in both H4IIE/ND and H4IIEins/ND cells. Electron microscopy revealed insulin storage vesicles in the H4IIE/ND and H4IIEins/ND cell lines. Regulated secretion of insulin to glucose (0–20 mmol/L) was seen in the H4IIEins/ND cell line. The H4IIEins/ND cells were transplanted into diabetic immunoincompetent mice, resulting in normalization of blood glucose. This data shows that the expression of *NeuroD1* and insulin in liver cells may be a useful strategy for inducing islet neogenesis and reversing diabetes.

## 1. Introduction

Diabetes mellitus is a chronic metabolic disorder of multiple etiologies [1,2]. The condition is characterized by chronic hyperglycaemia caused by immune-mediated beta (β) cell elimination or defects in insulin secretion and/or insulin action [1]. According to the International Diabetes Federation (IDF) [3], 415 million people have diabetes, and estimate that one person will die from diabetes-related conditions every six seconds. By 2040, it is estimated that 642 million people will have diabetes.

Diabetes is classified into three categories: type 1 diabetes mellitus (T1D), type 2 diabetes mellitus (T2D), and gestational diabetes [1]. T1D is caused by an absolute insulin deficiency, secondary to the autoimmune destruction of the insulin-secreting pancreatic β-cells [1]. T2D is characterized by insulin deficiency or insulin secretory defects, either with or without insulin resistance, and may be treated with oral medications, lifestyle, and dietary modifications; however, a large proportion of patients become insulin-dependent due to β-cell dysfunction. Gestational diabetes is characterized by hyperglycaemia caused by the inability to compensate for increased insulin demands during pregnancy [1].

Tight glucose control, through intensive insulin therapy, in insulin-dependent individuals, delays, but does not eliminate, complications such as nephropathy, retinopathy, cardiovascular disorders, and various neurological problems, which greatly increase mortality and morbidity [4]. β-Cell replacement is a promising approach for the treatment of diabetes; however, transplantation of pancreatic tissue is limited by a shortage of donors and the need for lifelong immunosuppression, which carries detrimental side effects. Thus, alternative therapeutic strategies must be developed. Gene therapy is one such strategy. The production of an insulin-secreting β-cell line, which is either resistant to recurrent autoimmunity, or able to modulate the immune system to induce tolerance, is required to reverse insulin-dependent diabetes. The importance of transcription factors in the regulation of cell fate during embryonic development has been widely demonstrated. The budding of the pancreas from the endoderm layer requires expression of the pancreatic transcription factor, *Pdx-1*. Subsequent pancreatic organogenesis is critically dependent upon the spatial and temporal expression of *Pdx-1*, and other pancreatic transcription factors [5]. *NeuroD1* is expressed immediately downstream of *Neurog3* in all pancreatic endocrine progenitors and is maintained in all later stages of β-cell development. It is also an activator of the insulin gene [6]. The knockout studies of *NeuroD1* in mice [7] resulted in reduction in the number of pancreatic β-cells and indicate the importance of *NeuroD1* in regulating the proliferation of endocrine cell lineages.

Due to the common endodermic origin of the liver and pancreas in the embryo [5], the ability to transdifferentiate tissue from liver to pancreas has been examined to a greater extent than other tissue types [8]. One approach is the delivery of β-cell transcription factors to the liver to generate the production of insulin-producing cells [9,10,11,12,13]. Our laboratory, and others, have shown that the ability of liver cells to both store and secrete insulin and undergo pancreatic differentiation is linked to the expression of β-cell transcription factors [8,9,10,11,12,13,14,15,16,17,18]. We had previously observed that expression of the insulin transgene in a human liver cell line, Huh7, which endogenously expresses β-cell transcription factors, resulted in pancreatic transdifferentiation with the formation of insulin storage granules and regulated secretion of insulin to glucose. After transplantation into diabetic non obese/severe combined immunodeficiency (NOD/*scid*) mice, hyperglycaemia was reversed [11]. We have also observed that the delivery of furin-cleavable human insulin (INS-FUR) directly to the livers of chemically-diabetic rats, spontaneously-diabetic NOD mice, and pancreatectomized pigs, resulted in the spontaneous expression of β-cell transcription factors, secretory granule biogenesis, reversal of diabetes, and normal glucose tolerance [16,17,18].

In the current study, we used a bicistronic retroviral vector to deliver either the INS-FUR gene alone, or the INS-FUR gene [19] and the rat *NeuroD1* gene together, to the rat liver cell line, H4IIE which, like normal primary hepatocytes, does not express the key β-cell transcription factors, to determine if the storage of insulin and pancreatic transdifferentiation could be induced. There are many choices of delivery of transgenes into cells [20]; however, viral vectors remain the most efficient way. A retroviral vector was used in this model system as it allows efficient integration of transgenes into the host genome, with long term expression and selection of stable clones possible via selection in G418 [21]. INS-FUR was used, rather than transduction with the rat insulin gene, in order to distinguish transgene and endogenous rat insulin gene expression levels. Additionally, normal liver cells do not have the insulin proconvertase enzymes that cleave proinsulin to insulin and c-peptide; hence, in this system mature insulin is produced from the INS-FUR construct following cleavage with furin, which is expressed in liver cells.

Briefly, the results of the study showed that the expression of INS-FUR together with the β-cell transcription factor *NeuroD1* in the H4IIEins/ND cell line had a synergistic effect leading to pancreatic transdifferentiation, storage of insulin in granules, regulated insulin secretion to glucose (and other β-cell secretogogues), and expression of a number of β-cell transcription factors and pancreatic hormones and ultimate reversal of diabetes. By comparison, expression of INS-FUR alone resulted in constitutive expression of insulin (H4IIEins), and *NeuroD1* alone (H4IIE/ND) resulted in expression of β-cell transcription factors together with some pancreatic hormones; however, insulin storage was low and no glucose-regulated insulin secretion was detected.

## 2. Results

### 2.1. Insulin Secretion and Storage

In order to determine if the transduced cell lines stored and secreted human and/or rat insulin, culture supernatants and acid-ethanol extracts were examined using specific commercial ELISA kits.

It can be seen from Table 1 that, as expected, H4IIE cells transduced with the empty vector (H4IIE-EV) did not store or secrete either human or rat insulin. H4IIEins cells secreted human insulin, but did not store any insulin. H4IIEins cells did not secrete or store rat insulin. By comparison H4IIE/ND and H4IIEins/ND cells stored and secreted both human and rat insulin following transduction, however H4IIEins/ND cells stored and secreted significantly higher amounts of both human and rat insulin compared to other cell lines. In comparison, the mouse β-cell line, MIN6 [22] which was used as a positive control in the electron microscope studies, stored 1678.0 ± 215.7 pmol/mouse insulin/5 × 10^6^ cells.

**Figure 1:**
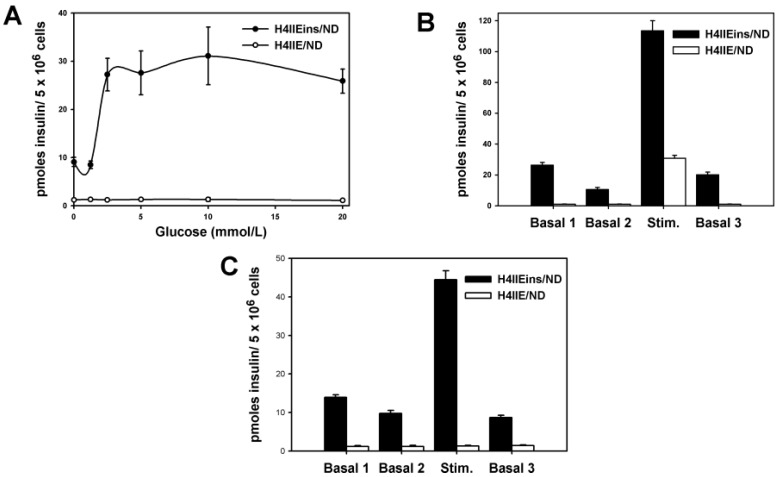
Insulin secretion from H4IIE/ND and H4IIEins/ND cells: (**A**) dose response curve to increasing concentrations of glucose, 0–20 mmol/L; (**B**) response to 10 mmol/L theophylline; and (**C**) 10 mmol/L calcium. Cells were incubated in the basal medium for two consecutive 1 h periods (Basal 1 and 2) before exposure to the stimulus for 1 h, followed by a third basal period (Basal 3). Values are expressed as means ± SE (*n* = 6).

The effect of three common β-cell stimuli (glucose, calcium, and theophylline) on the insulin secretion of the H4IIEins, H4IIE/ND, and H4IIEins/ND cells was determined. H4IIEins cells did not secrete increased amounts of insulin in response to stimulation with glucose, calcium, or theophylline. In response to varying concentrations of glucose (0–20 mmol/L), a dose-response curve for human insulin secretion was generated for H4IIEins/ND cells (Figure 1A). Whilst glucose-responsiveness started at a slightly lower concentration than seen in normal pancreatic islet cells, the secretion curve generated by H4IIEins/ND cells approached normal physiological levels. H4IIE/ND cells were not responsive to a glucose stimulus (Figure 1A). It was not possible to detect rat insulin secretion by static incubation, most likely because the levels were too low to detect using the assay system available.

A >20-fold and a six-fold stimulation of human insulin secretion above basal levels was detected when 10 mmol/L theophylline was applied as a stimulus to H4IIEins/ND cells and H4IIE/ND cells, respectively (Figure 1B). H4IIEins/ND cells showed a 4.5-fold increase in basal insulin secretion to a stimulus of 10 mmol/L calcium; however, H4IIE/ND cells were not responsive to the calcium stimulus (Figure 1C).

### 2.2. Transmission Electron Microscopy of Insulin Storage Granules

**Figure 2:**
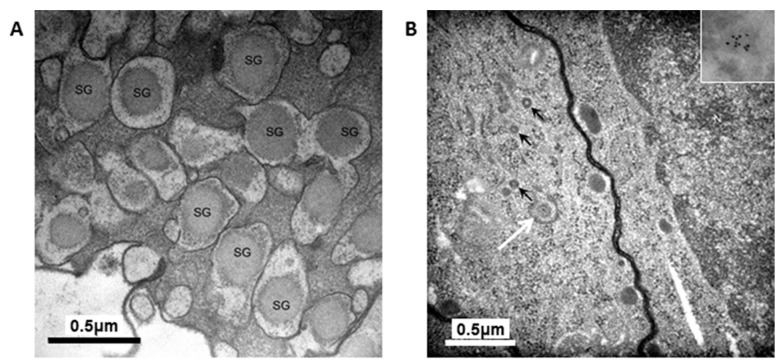

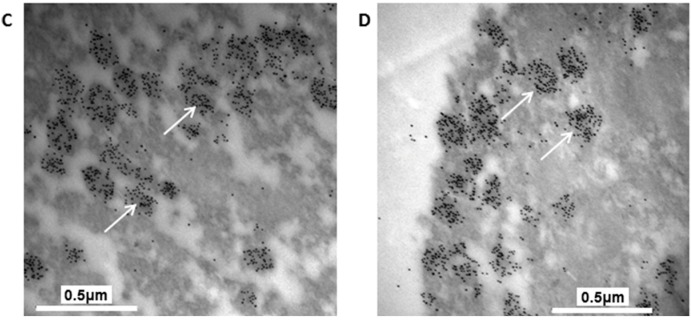
Transmission electron micrographs of: (**A**) H4IIEins/ND cells showing secretory granules (SG) surrounded by a pale halo; (**B**) H4IIE/ND cells showing immature secretory granules (marked with black arrows) and one larger secretory granule with a pale halo (marked by white arrow). The insert shows an immunoelectron micrograph (IEM) of a granule labelled with 10 nm gold particles; (**C**) IEM of H4IIEins/ND cells; and (**D**) MIN6 cells showing secretory granules labelled with 10 nm gold particles to show where insulin was stored in the cells (selected granules marked with arrows).

To determine if secretory granules were present in H4IIE/ND and H4IIEins/ND cells transmission electron microscopy (TEM) and immunoelectron microscopy (IEM) were performed. TEM revealed the presence of granules in the cytoplasm (270–330 nm in diameter) in H4IIEins/ND cells (Figure 2A), which were of a similar appearance and dimension to those in pancreatic β-cells [23]. Few cytoplasmic granules of this size were found in the H4IIE/ND cells (Figure 2B), although some smaller granules (150 nm diameter) were present, in accordance with the low amount of insulin that was stored by this cell line. The IEMs indicated that both H4IIE/ND and H4IIEins/ND cells stored insulin within granules in the cytoplasm of the cells (Figure 2B insert, C). Further, immunogold labelling revealed that the insulin-containing granules in H4IIEins/ND cells were of a similar size and density to those observed in the mouse β-cell line, MIN6 [21] (Figure 2D).

### 2.3. Reversal of Diabetes in Non-Obese Diabetic/Severe Combined Immunodeficiency (NOD/Scid) Mice

**Figure 3:**
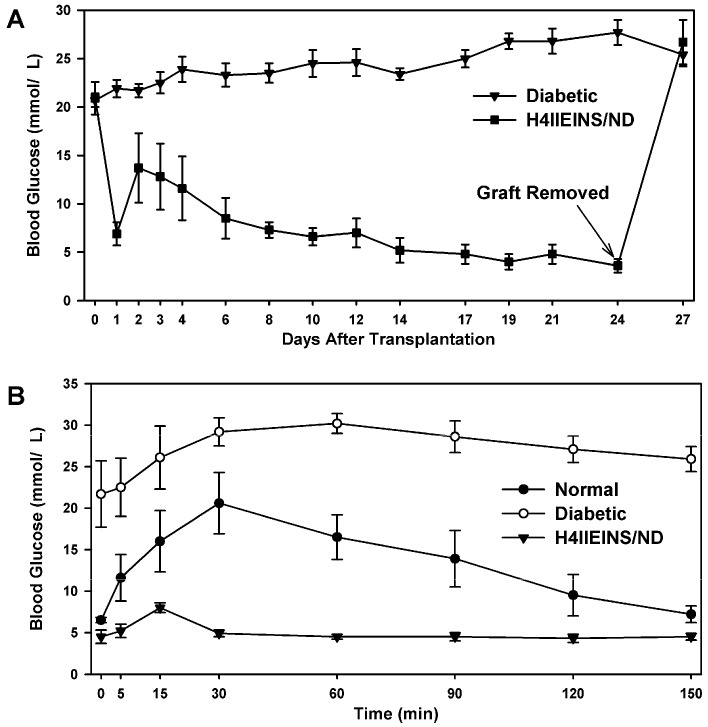
Reversal of diabetes in NOD/*scid* mice after transplantation of H4IIEins/ND cells: (**A**) blood glucose levels of NOD/*scid* mice following transplantation with H4IIEins/ND cells, together with diabetic controls. Grafts of H4IIEins/ND cells were removed at 24 days; and (**B**) intraperitoneal glucose tolerance test in mice following transplantation with H4IIEins/ND cells, together with normal (non-diabetic) and diabetic controls. Values are expressed as mean ± SEM (*n* = 7).

To determine if H4IIEins/ND cells could reverse diabetes in NOD/*scid* mice 1 × 10^7^ cells were transplanted subcutaneously. Transplanted H4IIEins/ND cells combined into a mass at the site of injection and grafts could be seen under the skin 1–2 weeks post-transplantation. All seen diabetic mice that were transplanted with H4IIEins/ND cells reverted to normoglycaemia (blood glucose 4.5–5.0 mmol/L) within 14 days. Blood glucose levels of animals transplanted with H4IIEins/ND cells remained normoglycemic until removal of the graft (day 24 post-transplantation), which was followed by an immediate reversion to hyperglycaemia (Figure 3A). On removal, the grafts were approximately 2–3 mm in diameter. These results indicated that reversal of hyperglcemia was attributable to the transplanted H4IIEins/ND cells and not to the regeneration of pancreatic β-cells.

The fasting blood glucose levels of mice following transplantation with H4IIEins/ND cells were not significantly lower than untreated normal control animals (Figure 3B). During the intraperitoneal glucose tolerance tests (IPGTTs), the glucose dose response curves for mice transplanted with H4IIEins/ND cells peaked at significantly lower blood glucose concentrations (8.0 ± 0.6 mmol/L), as compared to normal untreated animals (20.6 ± 3.7 mmol/L), and by 60 min blood glucose concentrations had returned to normal levels (4.5 ± 0.3 mmol/L glucose).

### 2.4. Immunohistochemistry of Islet Hormones in Transplanted H4IIEins/ND Cells

**Figure 4:**
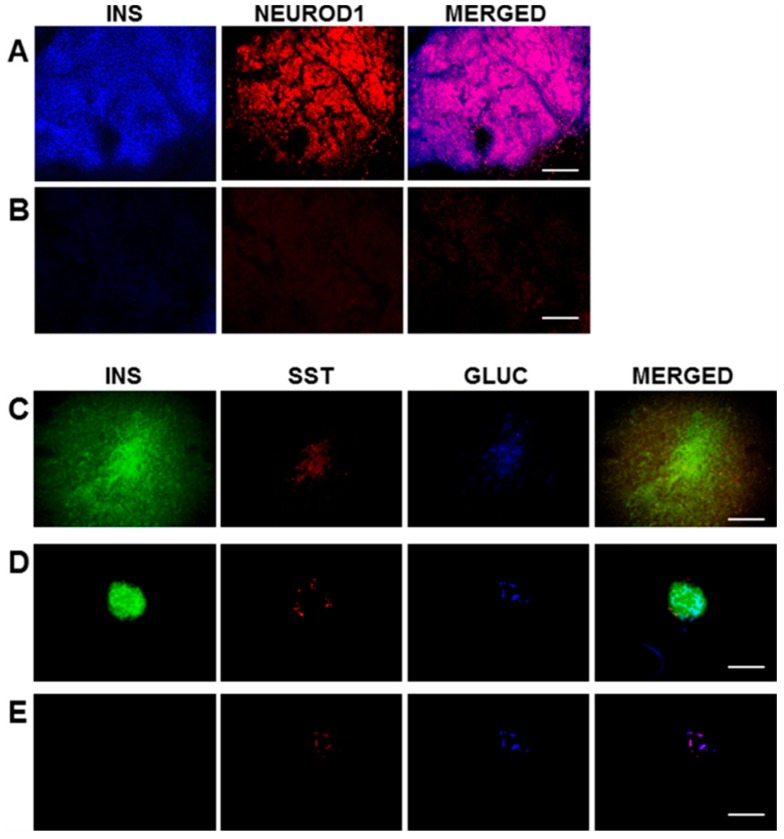
Expression of pancreatic hormones and NEUROD1 in grafts from NOD/*scid* mice following transplantation of H4IIEins/ND cells and diabetes reversal. Photomicrographs of double-stained anti-insulin and anti-NEUROD1 of (**A**) H4IIEins/ND cells; and (**B**) tissue near graft (bar = 80 µm); (**C**–**E**): Photomicrographs of triple anti-insulin (INS), anti-glucagon (GLUC) and anti-somatostatin (SST) staining of (**C**) H4IIEins/ND graft; (**D**) normal mouse pancreas; and (**E**) diabetic mouse pancreas (bar = 80 µm). Original magnification 200×.

After removal at day 24, grafts of H4IIEins/ND cells were examined for double-staining of insulin and NEUROD1 and triple-staining for the endocrine hormones insulin, glucagon, and somatostatin. Extensive staining for human insulin and NEUROD1 was seen in H4IIEins/ND grafts removed from animals (Figure 4A). The percentage of cells that stained positive for insulin, NEUROD1 and insulin + NEUROD1 was 50 ± 1.0, 56.8 ± 2.0 and 44 ± 1.8 (*n* = 10 sections), respectively. The remainder of the graft was composed of vascular and connective tissue. We examined pancreatic hormone-producing cells in the H4IIEins/ND grafts. Tissue close to the graft was negative for both insulin and NEUROD1 (Figure 4B). Figure 4C shows staining for insulin (52.8% ± 10%), glucagon (0.3% ± 0.1%) and somatostatin (3.8% ± 1.1%), with a small number of cells being positive for all three hormones (0.9% ± 0.4%) (*n* = 10 sections). Figure 4D shows a large amount of staining for insulin in pancreatic islets from non-diabetic NOD/*scid* mice. However, pancreatic β-cells were rarely seen in diabetic animals that were transplanted with H4IIEins/ND cells (Figure 4E).

### 2.5. Expression of β-Cell Related Genes in H4IIEins and H4IIEins/ND Cells

**Figure 5:**
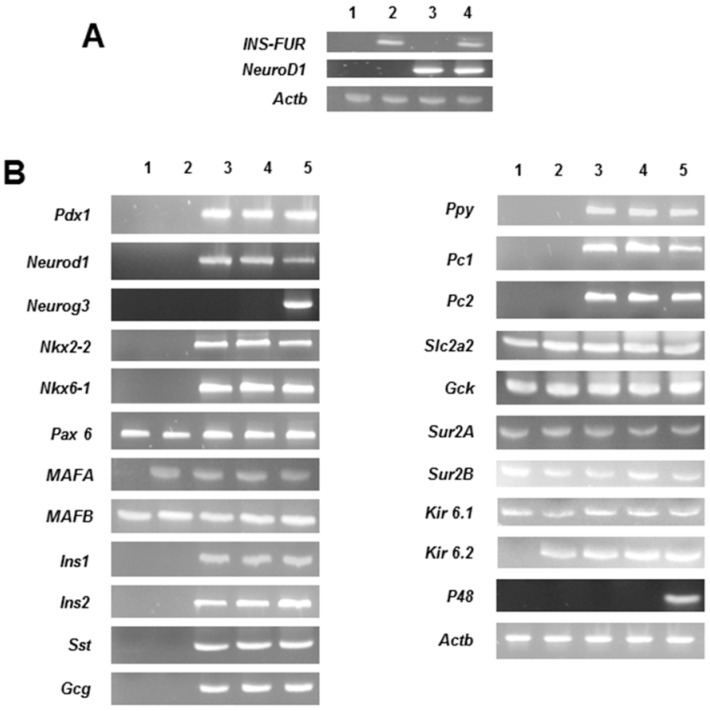
Pancreatic hormones and β cell transcription factors expressed in rat liver cell lines. (**A**) reverse transcription polymerase chain reaction (RT-PCR)analysis of cell lines for INS-FUR, *NeuroD1* and β-actin (*Actb*): H4IIE (lane 1), H4IIEins (lane 2), H4IIE/ND (lane 3), and H4IIEins/ND (lane 4); (**B**) RT-PCR analysis for: β cell transcription factors (*Pdx1*, *Neurog3*, *NeuroD1*, *Nkx2-2*, *Nkx6-1*, *Pax6*, *MAFA*, *MAFB*); the rat pancreatic endocrine hormones insulin 1 and 2 (*Ins1*, *Ins2*), glucagon (*Gcg*), somatostatin (*Sst*), and pancreatic polypeptide (*Ppy*); GLUT2 (*Slc2a2*) and glucokinase (*Gck*); insulin proconvertase PC1 (*PC1*) and PC2 (*PC2*); the exocrine marker *p48*; the potassium channel proteins SUR 2A, SUR2B, Kir 6.1 and Kir 6.2 and β-actin in H4IIE (lane 1), H4IIEins (lane 2), H4IIE/ND (lane 3), H4IIEins/ND (lane 4), and rat pancreatic tissue (lane 5).

To determine if the pancreatic transdiffereniation seen in H4IIE/ND and H4IIEins/ND cells is a result of induced expression of β-cell transcription factors, reverse transcription polymerase chain reaction (RT-PCR) and real time quantitative polymerase chain reaction (RT-qPCR) was performed. As expected, H4IIEins and H4IIEins/ND cells expressed INS-FUR and H4IIE/ND, and H4IIEins/ND cells expressed *NeuroD1* (Figure 5A). Expression of *NeuroD1* in both the H4IIE/ND and H4IIEins/ND cells stimulated expression of both upstream (*Pdx1*) and downstream (*NeuroD1*, *Pax4*, *Nkx2.2*, *Nkx6.1*) β-cell transcription factors. *Neurog3* was not detected in any of the cell lines and *Pax 6* was detected in all cell lines, including the parent cell line, H4IIE. Rat insulin 1 and 2, somatostatin, glucagon, and pancreatic polypeptide were expressed in the H4IIE/ND and the H4IIEins/ND cells. *MafA* and Kir 6.2 were expressed in all cell lines, with the exception of the H4IIE cells. *MafB* and the channel proteins SUR2A, SUR2B, and Kir 6.1 were expressed in all cell lines, as were rat glucokinase and GLUT2. The exocrine factor, p48, was not expressed in any of the cell lines (Figure 5B).

Examination of the expression levels of several key β-cell transcription factors by qRT-PCR indicated that there was an 8.9 ± 0.5-fold increase in *Pdx-1*, a 6.5 ± 0.4-fold increase in *NeuroD1*, a 9.6 ± 1.7 increase in rat *Nkx2.2*, a 4.5 ± 0.9-fold increase in rat insulin 1, and a 2.3 ± 0.4-fold increase in rat insulin 2 expression in H4IIEins/ND cells (*n* = 6), as compared to H4IIE/ND cells. By comparison, there were no differences in the expression levels of the other factors examined between the two cell lines (Figure 5B).

## 3. Discussion

In the current study, the rat liver cell line, H4IIE, which, like primary hepatocytes, does not express major β-cell transcription factors, was engineered to express INS-FUR together with rat *NeuroD1* (H4IIEins/ND cells). These cells developed insulin storage granules and, following transplantation, reversed diabetes in NOD/*scid* mice (Figure 3A). The H4IIEins/ND cells expressed β-cell transcription factors and exhibited regulated insulin secretion. In the presence of *NeuroD1* alone (H4IIE/ND cells), β-cell transcription factors were expressed, but insulin storage was very low and glucose-responsive insulin secretion was absent. These results support our previous findings [14,15,16,17,18] that, under certain conditions, the dual expression of insulin and a β-cell transcription factor is superior to the expression of insulin or the β-cell transcription factor alone [9,10,11,12,13,24,25,26].

In a pancreatic β-cell, regulated physiological secretion of insulin is facilitated by the storage of insulin in secretory granules in the cytoplasm. Previous studies have shown that the development of secretory granules in liver cells can be induced by the expression of various β-cell transcription factors. Ferber *et al.* [9] delivered *Pdx1* to mouse liver tissue, via recombinant adenovirus-mediated transfer, and showed that expression of *Pdx-1* in the livers of diabetic mice resulted in insulin expression and secretion with restoration of normoglycaemia due to hepatic insulin production. However, normoglycaemia was transient (only sustained for eight days) and exocrine transdifferentiation of the liver resulted in the development of hepatitis. Kojima *et al.* [11] observed similar exocrine differentiation after delivery of *Pdx1* to mouse livers, which was likely attributable to the use in the study of a powerful ubiquitously expressed elongation factor-1α promoter, resulting in constitutive high level expression of *Pdx-1*. Kojima *et al* [11] also incorporated *NeuroD1* together with βcellulin within an adenoviral vector and transduced the livers of streptozotocin (STZ)-treated diabetic mice. It was found that the expression of *NeuroD1*-βcellulin reversed diabetes and maintained normoglycaemia, with associated up-regulation of both upstream and downstream β-cell transcription factors, including *Pdx1*, *Pax6*, *Pax4*, *Nkx2.2*, and *Nkx6.1*, as seen in the current study. This up-regulation of *Pdx1* may be a method (as suggested by Kojima *et al.* [11]) by which *NeuroD1*, in a “feed-forward fashion, induces neogenesis of all major hormone producing cells in the newly formed islets”. This same mechanism may have occurred in the H4IIE/ND and H4IIEins/ND cell lines following transduction with *NeuroD1*. In the Kojima study insulin storage levels were low [11], as observed in the H4IIE/ND cells. By comparison, the insulin content of the H4IIEins/ND cells was not significantly different to the MIN6 β-cell line. The H4IIEins cell line that expressed no β-cell transcription factors simply secreted insulin in a constitutive fashion with no detectable storage.

The granules in the H4IIEins/ND cells were similar in size and appearance to those seen in normal pancreatic β-cells, and to those observed in Melligen cells and NOD mice that have been engineered to express insulin in their livers [15,17]. The granules in the H4IIEins/ND cells were typical of mature β-cell granules which have an electron-dense core and a granule-limiting membrane with the presence of a halo around the core [23]. The amount of immunogold staining in H4IIEins/ND cells was comparable to that seen in MIN6 cells, which correlated with the similarly large amount of insulin that was stored in the two cell lines. By comparison, the granules of H4IIE/ND cells were sparse and looked immature in appearance. Only small numbers of granules were detected in H4IIE/ND cells, which correlates with the comparably low storage of insulin. Extraction of the cells with acid/ethanol indicated that both rat and human insulin was stored in the granules of H4IIE/ND and H4IIEins/ND cells, with a significantly greater proportion of human insulin.

The expression of the β-cell transcription factor, *NeuroD1*, most likely stimulated the expression of the network of transcriptional factors required for pancreatic neogenesis in H4IIE/ND and H4IIEins/ND cells, including upstream expression of *Pdx1* and the other major factors involved in islet development, including *Nkx2.2* and *Nkx6.1*. The net result was the expression of the rat pancreatic hormones insulin, glucagon, somatostatin, and pancreatic polypeptide. *MafA* was expressed in all transduced cell lines, including H4IIEins cells, and whilst the role of *MafA* in the development of the pancreatic β-cells has not been fully delineated, it is thought to be a mediator of insulin gene transcription [27]. In contrast to our previous studies [16,17], the proconvertases, PC1 and PC2, were also expressed in both H4IIE/ND and H4IIEins/ND cells. *MafB* was expressed in all cell lines, which was not unexpected as it has been reportedly expressed in liver tissue [17,27]. The expression of the potassium channel subunits (SUR1, SUR2, Kir 6.1) in all the cell lines and the expression of Kir 6.2 in all the transduced cell lines is consistent with observations that these channels are expressed in liver tissue [27,28,29]. Previous studies using other cell lines, which have been induced to secrete insulin in a regulated fashion, have shown that responsiveness to glucose, and other pharmacological stimuli, occurs through the activation of ATP-sensitive potassium and calcium channels [28,29,30], which may be the case in the current study, but this would require confirmation. The exocrine marker, p48, was not expressed in any of the transduced cells, thereby preventing exocrine transdifferentiation which would have caused cell destruction, as reported in other studies [9,11].

The H4IIEins/ND cell line commenced increased secretion of insulin to glucose at 2.5 mmol/L, which is less sensitive than pancreatic β-cells, which respond in the 4–5 mmol/L range, but identical to HEPGins/g and Huh7ins cells [14,31] that were engineered to secrete insulin in the presence of the endogenous expression of β-cell transcription factors. In the Huh7ins cells [14,15], the suboptimal response to glucose was a result of higher expression levels of hexokinase, as compared to glucokinase. When islet glucokinase was expressed in the Huh7ins cells, the resulting Melligen cell line responded to glucose in the physiological range [15]. When transplanted into diabetic NOD/*scid* mice, the H4IIEins/ND cells achieved normoglycaemia; however, normal glucose tolerance was not observed, with IPGTTs showing peak responses at a much lower value than for normal animals, which was similar to the result observed using Huh7ins cells [14]. This anomaly was corrected in the Melligen cell line when the glucokinase: the hexokinase ratio was adjusted in favor of glucokinase (as is observed in pancreatic β-cells) and normal glucose tolerance was established. The H4IIEins/ND cells also responded well to a calcium and theophylline stimulus, whilst H4IIE/ND cells showed a small response to calcium alone. In an attempt to understand why expression of INS-FUR and *NeuroD1* resulted in significantly increased secretion of insulin to glucose (and the other secretogogues tested), we performed RT-qPCR to compare expression levels of the β-cell transcription factors and other genes. Compared to the H4IIE/ND cells, the H4IIEins/ND cells showed a significant up-regulation of expression of several key β-cell transcription factors, *Pdx1*, *NeuroD1*, and *Nkx2.2*, and the rat insulin genes 1 and 2, which likely produced a more mature β-cell phenotype, characterized by a greater number of insulin secretory granules and regulated glucose-stimulated insulin release. However, the reason why the dual expression of INS-FUR and *NeuroD1* has resulted in extensive storage of the INS-FUR and regulated secretion of human, rather than rat insulin, to glucose is yet to be elucidated. The expression of *NeuroD1* undoubtedly resulted in secretory granule biogenesis, and because INS-FUR is overexpressed in the H4IIEins/ND cells the cells likely produced increased amounts of human, as compared to rat insulin. Feedback mechanisms are probably activated when a liver cell produces large amounts of insulin in the presence of β-cell transcription factors; however, these are not currently identified. Indeed, the pathways of β-cell transcription factors and their interactions are continually being elucidated and updated [5] and ongoing studies in our laboratory, and others, will likely lead to greater understanding of the pathways in the future.

The H4IIEins/ND cell line is not a clinically relevant system as, most importantly, it is derived from a tumorigenic rat liver cell line. Additionally, whilst a retroviral vector [32] was utilized in this model system as a tool to investigate the dual expression of *NeuroD1* and human insulin in a liver cell, there are concerns with the clinical application of these vectors in that they may induce oncogene expression when the residual viral genome is integrated close to the location of an oncogene region in the host genome [33]. Secondly, they only transduce dividing cells [34]. More appropriate vectors for clinical application would be lentiviral vectors that can transduce quiescent cells without a significant immune response [17,34] or adeno-associated vectors (AAV), which allow site-specific integration of transgenes into the host chromosome and new developments describe AAV cassettes that specifically transduce human cells [35].

## 4. Materials and Methods

### 4.1. Vectors

The INS-FUR gene [19] (gift from Genetech, San Franscico, CA, USA) and/or the rat *NeuroD1* gene were cloned into the retroviral vector *pLXSN* [21] (gift from G. Marshall, Children’s Cancer Institute, Randwick, NSW, Australia) between the *HpaI* and *XhoI* sites. The bicistronic segment containing the genes was constructed in the *pGEM2*-derived vector *pSXLC* in which the internal ribosomal entry site had been inserted into the modified multi-cloning site of the vector [32] (gift from T. Friedmann, Centre for Molecular Genetics, University of California, San Diego, CA, USA). All molecular manipulations were approved by the University of Technology Sydney (UTS), Biosafety Committee, (Protocol 2011-23-R-GC). A schematic representation of the vectors can be found in Figure S1. Retroviral vector constructs containing INS-FUR and/or *NeuroD1* or empty vector were introduced into the amphotrophic packaging cell line, PA317. The medium from the PA317 cell line was used to transduce the H4IIE cells. Stable clones were selected in G418 (1.4 µg/mL, Life Technologies, Mulgrave, VIC, Australia) and expanded for further analysis: H4IIE-EV (empty vector), H4IIEins cells (INS-FUR gene alone), H4IIE/ND (*NeuroD1* gene alone), and H4IIEins/ND (INS-FUR and *NeuroD1* genes). At least 10 clones were isolated from each group and the cell line with the highest level of insulin storage was selected for further experimentation.

### 4.2. Cell Culture

The amphotrophic cell line, PA317 was obtained from G. Marshall (Children’s Cancer Institute, Randwick, NSW, Australia) and the H4IIE parent cell line was obtained from the American Type Culture Collection (Manassas, VA, USA). Cell lines were cultured in Dulbecco’s modified Eagle’s medium (DMEM, Trace Biosciences, Castle Hill, NSW, Australia), containing 4.5 g/L glucose, supplemented with 44 mmol/L sodium bicarbonate and 10% fetal calf serum (FCS, Trace Biosciences) and 5% CO_2_/37 °C in a humidified incubator. MIN6 cells [17] were grown in the presence of 15% *v*/*v* FCS. H4IIEins cells, H4IIE/ND and H4IIEins/ND were grown in the presence of 10% *v*/*v* FCS and G418 (1.4 µg/mL) to maintain selective pressure.

### 4.3. Insulin Secretion and Storage

To determine if the cell lines stored insulin, 5 × 10^6^ cells were re-suspended in 500 NOD/*scid* of 0.18 N HCl in 70% ethanol for 48 h at 4 °C. For the measurement of insulin content, samples were diluted (1:10 or 1:100) and insulin levels were measured using an ELISA kit (EZHI-14K, Merck Millipore, MA, USA), which had no cross-reactivity with human proinsulin or rat insulin. For the measurement of rat insulin the EZRMI-12K kit (Merck Millipore) was used, which had no cross-reactivity with human insulin. To determine acute secretion of insulin to stimuli, tissue culture wells were washed with basal medium (phosphate buffered saline [PBS] containing 1 mmol/L CaCl_2_ and supplemented with 20 mmol/L HEPES and 2 mg/mL bovine serum albumin [BSA], 2.8 mmol/L glucose, (unless otherwise stated), pH 7.4. Monolayers were incubated in the basal medium for two consecutive 1 h periods to obtain a basal insulin reading. Monolayers were then exposed to stimuli for a further period of 1 h. The effect of increasing concentrations of glucose (from 0 to 20 mmol/L), theophylline (10 mmol/L) and calcium (10 mmol/L) on acute insulin secretion was assessed by ELISA.

### 4.4. Transplantation of Engineered Cells

Male NOD/*scid* mice were sourced from the Australian Research Council (ARC) Facility (Perth, WA, Australia). All animal studies were performed according to the guidelines of the National Health and Medical Research Council of Australia principles of animal care and regulations of the ARC, following approval by the Animal Care and Ethics Committee at UTS (Protocol 2008-37). Injection of STZ, (170 mg/kg) induced diabetes in the mice. After treatment, animals were monitored for blood glucose and body weight change. Diabetes was defined as three consecutive blood glucose readings >15mmol/L. Rat pancreatic tissue was sourced from male Wistar rats (Gore Hill Research Laboratories, Sydney, NSW, Australia) with ethics approval as above.

H4IIEins/ND cells (1 × 10^7^) (*n* = 6) were aspirated into a syringe with a 21 g needle, and injected subcutaneously above the right scapula of the mice. Weight and blood glucose levels were monitored every 1–3 days. No insulin was given to the mice during the experiment. Intraperitoneal glucose tolerance tests (IPGTT) (2 g glucose/kg after a 6 h fast) were carried out on the animals transplanted with H4IIEins/ND cells prior to removal of the grafts together with diabetic and normal control animals.

### 4.5. Microscopic Analysis

For immunofluorescence, frozen sections (6–8 μm) of pancreas and transplanted H4IIEins/ND cells were cut. For the triple staining of insulin, somatostatin, and glucagon, the primary antibodies were mouse anti-human insulin (1:100 BioGenex, Fremont, CA, USA), goat anti-somatostatin, and goat anti-glucagon (1:200, Santa Cruz, CA, USA). The secondary antibodies used were anti-mouse IgG for insulin, and anti-goat IgG for somatostatin and glucagon (1:200, Vector, Laboratories, Burlingame, CA, USA). The fluoresceins used were avidin D, rhodamine avidin D, and AMCA avidin D (1:200, Vector Burlingame, CA, USA). The fluorescein M.O.M kit (FMK–220, Vector Laboratories, Burlingame, CA, USA) was used for staining, following the manufacturer’s instructions. The avidin/biotin blocking kit (sp-200, Vector, CA, USA) was used, as per the manufacturer’s instructions, between applications of the different antibodies during the triple-staining procedure. For double-staining of insulin and *NeuroD1*, the sections of transplanted cells were incubated (1 h) with mouse anti-human insulin (1:100 BioGenex, Fremont, CA, USA) and goat anti-*NeuroD1* (Santa Cruz, CA, USA), which were diluted 1:100 in 10% donkey serum at room temperature (RT). The secondary antibodies were subsequently incubated at RT (1 h) in the case of insulin, with FITC-conjugated donkey anti-mouse IgG, or rhodamine red conjugated donkey anti-goat IgG for *NeuroD1* (Jackson ImmunoReseach, Westgrove, PA, USA), which was diluted 1:100 in 10% donkey serum and incubated for 1h at RT. Vectashield mounting medium (Vector Labratories, Burlingame, CA, USA) was used to mount sections which were examined under an Olympus BX60 fluorescent microscope. Analysis of the data was performed using the Olympus Image Pro 6.2 software (Media Cybernetics, Inc., Rockville, MD, USA).

For electron microscopy, cells were fixed and processed as previously described [17]. For insulin IEM, a post-embedding immunogold procedure was used. Cells were embedded in LR white and labelling procedures were performed as previously described [17].

### 4.6. RT-PCR and RT-qPCR Analysis of Gene Expression

For RT-PCR analyses, H4IIE, H4IIEins, H4IIE/ND, and H4IIEins/ND were snap-frozen in liquid nitrogen. Rat pancreas was used as the positive control. Total RNA was extracted with Trizol (Invitrogen/ ThermoFisher Scientific, Waltman, MA, USA) and samples were treated with DNase I (Invitrogen). Levels of expression of INS-FUR, other pancreatic hormones (rat insulin 1 and 2, glucagon, somatostatin, pancreatic polypeptide), liver glucokinase, GLUT2, the proconvertases, PC1 and PC2, several β-cell transcription factors (*Pdx-1*, *NeuroD1*, *Neurog3*, *Nkx2.2*, *Nkx6.1*, *Pax4*, *Pax6*), *MafA*, *MafB*, and the channel proteins, SUR2A, SUR2B, Kir6.1, and Kir 6.2, were determined.

cDNA was used as the template for real-time RT-qPCR using the SYBR GreenER qPCR Supermix (Invitrogen). The Eppendorf Realplex2 was the detection system. Real-time RT-qPCR reactions contained either primers specific to the reference gene, β-actin or the gene of interest (Table S1). Data was analysed using the 2^−∆∆t^ method: ∆∆*C*_T_ = (*C*_t, target_ − *C*_t, reference_)_control tissue_ − (*C_t_*_, target_ − *C*_t, reference_)_sample tissue_. The fold change in the expression of the target gene was normalised to β-actin and was relative to the expression in the control tissue. Means and standard error of the mean (SEMs) in fold changes in expression for each gene of interest were calculated. Oligonucleotide primer sequences can be found in Table S1.

### 4.7. Statistical Analysis

Data are represented as mean values ± SEMs. Comparisons between two groups were analysed using Student’s *t*-test. Comparisons between more than two groups were calculated using ANOVA (SPSS 17.0.0, SPSS Inc. 2008, Softonic, Barcelona, Spain). Repeated-measures analyses were used where it was appropriate. *p* Values <0.05 were considered to be statistically significant.

## 5. Conclusions

In conclusion, this study is the first to show increased insulin storage and secretion of insulin to glucose following the dual expression of *NeuroD1* and insulin in liver cells and gives promise that this gene combination delivered by a clinically applicable vector system is a viable strategy to ameliorate diabetes in insulin-dependent individuals.

## Figures and Tables

**Table 1:** Insulin secretion and storage from transduced H4IIE cell lines.

Cell Line	Human Insulin		Rat Insulin	
	Secretion	Storage	Secretion	Storage
H4IIE	ND	ND	ND	ND
H4IIE-EV	ND	ND	ND	ND
H4IIEins	0.5 ± 0.01	ND	ND	ND
H4IIE/ND	4.2 ± 0.9	65.3 ± 5.6	1.0 ±0.1	2.2 ± 0.4
H4IIEins/ND	32.1 ± 2.1 *	1475.4 ± 171.2 *	3.1 ± 0.9 *	6.3 ± 1.4 *

* Insulin secretion is expressed as pmol insulin/5 × 10^6^ cells/24 h and insulin storage as pmol/insulin/5 × 10^6^ cells. Cell lines were H4IIE (parent cell line), H4IIE-EV (empty vector), H4IIEins (INS-FUR gene alone), H4IIE/ND (rat *NeuroD1* gene alone), H4IIEins/ND (INS-FUR and rat *NeuroD1* genes); ND: not detectable. Asterisks represent a significantly higher value compared to other cell lines. Values are expressed as means ± SE (*n* = 3).

## Supplementary Materials

Supplementary materials can be found at http://www.mdpi.com/1422-0067/17/4/534/s1.

## Abbreviations

T1D	Type 1 diabetes
T2D	Type 2 diabetes
IDF	International Diabetes Federation
INS-FUR	Furin-cleavable human insulin
IPGTT	Intraperitoneal glucose tolerance test
ARC	Australian Research Council
NOD*/scid*	Non obese/severe combined immunodeficiency
STZ	Streptozotocin
TEM	Transmission electron microscopy
IEM	Immuno electronmicroscopy
UTS	University of Technology Sydney
AAV	Adeno-associated vector
