# Supplementary material for: Pancreatic Transdifferentiation and Glucose-Regulated Production of Human Insulin in the H4IIE Rat Liver Cell Line
Source: Int J Mol Sci. 2016 Apr 8;17(4):534. doi: 10.3390/ijms17040534 (PMC4848990; doi:10.3390/ijms17040534)
Supplement: Supplementary file 1 [file ijms-17-00534-s001.pdf]

Supplementary Materials: Pancreatic Transdifferentiation and Glucose-Regulated Production of Human Insulin in the H4IIE Rat Liver Cell Line

Binhai Ren, Chang Tao, Margaret Anne Swan, Nichole Joachim, Rosetta Martiniello-Wilks, Najah T. Nassif, Bronwyn A. O'Brien and Ann M. Simpson

Table S1. Oligonucleotide primers for RT-PCR and real-time RT-qPCR analysis.

Gene	Forward Primer (5′–3′)	Reverse Primer (5′–3′)
NEUROGEN3	CCGGATGACGCCAAACTTACA	ACACCAGTGCTCCCGGGAG
PAX6	AAGAGTGGCGACTCCAGAAGTTG	ACCACACCTGTATCCTTGCTTCAGG
NKX2.2	CACGCAGGTCAAGATCTG	TGCCCGCCTGGAAGGTGGCG
NKX6.1	ATGGGAAGAGAAAACACACCAGAC	TAATCGTCGTCGTCCTCCTCGTTC
β-ACTIN	CGTAAAGACCTCTATGCCAA	AGCCATGCCAAATGTCTCAT
PDX1	AAACGCCACACAAGGAGAAC	CTGTTATGGGACCGCTCAAG
RAT INSULIN 1	ATGGCCCTGTGGATGCGCTT	TAGTTGCAGTAGTTCTCCAGCT
RAT INSULIN 2	ATGGCCCGTGGGATCCGCTT	TGCCAAGGTCTGAAGGTCAC
NEUROD 1	GGACTTTCTTGCCTGAGCAGA	AACTCGGTGGATGGTTCGTGT
INS-FUR	AGCCTTTGTGAACCAACACC	CCAGTTGGTAGAGGGAGCAG
PC1	AATCCTGTAGGCACCTGGAC	GGAGTTTTTGGGTACCAGGA
PC2	GAGAGGAGACCTGAACATCA	CTTCGGCCACGTTCAAGTCTA
P48	GTCCCTGGAGCATTTTCCCG	CTGAGGAACTCTTACCTCCGC
GLUT2	GACACCCCACTCATAGTCACA	CAGCAATGATGAGAGCATGTG
GK	TGGATGACAGAGCCAGGATGG	ACTTCTGAGCCTTCTGGGGTG
GLUCAGON	GACCGTTTACGTGGCTGG	CGGTTCCTCTTGGTGTTCATCAAC
SOMATOSTATIN	GTTTCTGCAGAAGTCTCTGG	AGTTCTTGCAGCCAGCTTTG
PANCREATIC POLYPEPTIDE	TGAACAGAGGGCTCAATACGAAAC	AGACAGAAGGGAGGCTACAAATCC
MAF A	CGCAGGCCACCACGTGCGCTTGGAGGAG	CTGCGCTGGCGAGGGCTCCCGAGGGAAG
MAF B	CAACAGCTACCCACTAGCCA	GGCGAGTTTCTCGCACTTGA
SUR2A	GGCTCTGGGAGGCTCT	CATGTCCGCACGAACGAACGAG
SUR2B	CCTGGAGGTTCAGACTCTTGG	GCCTTAGGAAGCAGTGTTCTGTCCAT
KIR6.1	AGCCACTGACCTTGTCAACC	GGAGTCATGAATTGCACCTT
KIR6.2	CGTCACAAGCATCCACTCCT	CACCTGCATATGAATGGTGG

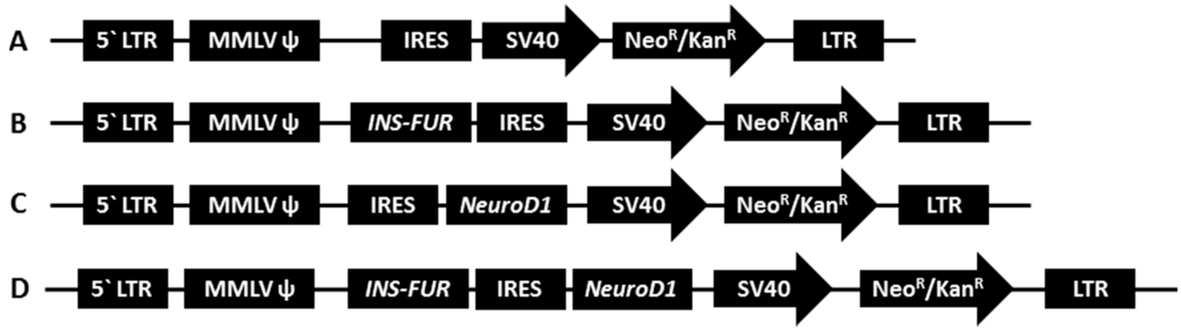

Figure S1. Schematic diagram of the vectors constructed from the retroviral vector *pLXSN* [21] used in this study. (**A**) *pLXSN* empty vector; (**B**) *pLXSN* containing INS-FUR; (**C**) *pLXSN* containing *NeuroD1*; (**D**) *pLXSN* containing INS-FUR and *NeuroD1*. LTR: long terminal repeat, Ψ: extended packaging signal, MMLV: Moloney murine leukemia virus, MCS: multi-cloning site, IRES: internal ribosomal entry site, Neo: Neomycin, Kan: Kanamycin, SV40: SV40 early promoter which controls the expression of the neomycin resistance gene.
